# Partial reduction of amyloid β production by β-secretase inhibitors does not decrease synaptic transmission

**DOI:** 10.1186/s13195-020-00635-0

**Published:** 2020-05-26

**Authors:** Tugce Munise Satir, Lotta Agholme, Anna Karlsson, Mattias Karlsson, Paul Karila, Sebastian Illes, Petra Bergström, Henrik Zetterberg

**Affiliations:** 1 Institute of Neuroscience and Physiology, Department of Psychiatry and Neurochemistry, the Sahlgrenska Academy at the University of Gothenburg, S-415 30 Gothenburg, Sweden; 2 Cellectricon AB, Neongatan 4B, S-431 53 Mölndal, Sweden; 3 Institute of Neuroscience and Physiology, Department of Psychiatry and Neurochemistry, The Sahlgrenska Academy at the University of Gothenburg, S-431 80 Mölndal, Sweden; 4 Clinical Neurochemistry Laboratory, Sahlgrenska University Hospital, S-431 80 Mölndal, Sweden; 5 Department of Neurodegenerative Disease, Institute of Neurology, University College London Queen Square, WC1N 3BG, London, UK; 6 UK Dementia Research Institute at UCL, WC1E 6BT, London, UK

**Keywords:** BACE inhibition, Synaptic transmission, Alzheimer’s disease, Amyloid beta, Beta-secretase

## Abstract

**Background:**

Alzheimer’s disease (AD) is the most common form of age-related neurodegenerative diseases. Cerebral deposition of Aβ peptides, especially Aβ42, is considered the major neuropathological hallmark of AD and the putative cause of AD-related neurotoxicity. Aβ peptides are produced by sequential proteolytic processing of APP, with β-secretase (BACE) being the initiating enzyme. Therefore, BACE has been considered an attractive therapeutic target in AD research and several BACE inhibitors have been tested in clinical trials, but so far, all have had negative outcomes or even led to worsening of cognitive function. AD can be triggered by Aβ years before the first symptoms appear and one reason for the failures could be that the clinical trials were initiated too late in the disease process. Another possible explanation could be that BACE inhibition alters physiological APP processing in a manner that impairs synaptic function, causing cognitive deterioration.

**Methods:**

The aim of this study was to investigate if partial BACE inhibition, mimicking the putative protective effect of the Icelandic mutation in the *APP* gene, could reduce Aβ generation without affecting synaptic transmission. To investigate this, we used an optical electrophysiology platform, in which effects of compounds on synaptic transmission in cultured neurons can be monitored. We employed this method on primary cortical rat neuronal cultures treated with three different BACE inhibitors (BACE inhibitor IV, LY2886721, and lanabecestat) and monitored Aβ secretion into the cell media.

**Results:**

We found that all three BACE inhibitors tested decreased synaptic transmission at concentrations leading to significantly reduced Aβ secretion. However, low-dose BACE inhibition, resulting in less than a 50% decrease in Aβ secretion, did not affect synaptic transmission for any of the inhibitors tested.

**Conclusion:**

Our results indicate that Aβ production can be reduced by up to 50%, a level of reduction of relevance to the protective effect of the Icelandic mutation, without causing synaptic dysfunction. We therefore suggest that future clinical trials aimed at prevention of Aβ build-up in the brain should aim for a moderate CNS exposure of BACE inhibitors to avoid side effects on synaptic function.

## Background

Alzheimer’s disease (AD) is the most common age-related form of dementia, and it is estimated that almost 50 million people are affected worldwide [1]. Until this day, there is no treatment available to stop or even slow down the disease process. Such treatments are urgently needed as the incidence is predicted to increase due to the aging population globally. Pathologically, AD is characterized by accumulation of amyloid β (Aβ) in extracellular senile plaques and tau in intracellular fibrillary tangles in the brain. Although the exact cause(s) of AD is still a matter of debate, most data suggest that cerebral accumulation of aggregated Aβ is triggering the disease process, with tau pathology being a downstream alteration [2, 3]. Therefore, treatments aimed at decreasing Aβ production or increasing Aβ clearance from the brain have been thoroughly investigated for more than a decade.

Aβ peptides are generated from the sequential cleavage of Aβ precursor protein (APP) by β- and γ-secretases [4]. Therefore, a multitude of drug candidates targeted at either β- or γ-secretase have been developed to treat AD [5]. γ-Secretase inhibitors and modulators were the first to be tested in clinical trials, but all the trials have had to be halted due to lack of efficacy or sometimes serious side effects [6]. γ-Secretase has many other biological substrates that could explain the negative effects and therefore the focus turned to β-secretase (BACE) inhibitors. There is a considerable amount of evidence regarding the involvement of BACE1 in AD pathogenesis. Increased protein levels and activity of BACE1 have been reported in the normal aging brain and to an even larger extent in the AD brain [7–9]. In addition, a mutation in *APP* resulting in increased BACE1 cleavage (called the Swedish mutation) results in increased Aβ production and a familial form of AD (FAD) [10]. On the contrary, the so-called Icelandic mutation in *APP* [11], which alters one amino acid at the BACE1 cleavage site of APP, reducing the ability of BACE1 to cleave APP by about 30% [12], is strongly protective against AD. Therefore, a wide variety of small molecules inhibiting BACE was developed and brought to clinical trials. Again, although these inhibitors successfully reduce Aβ production in both animals and humans [13, 14], they have been discontinued in phase II or III trials due to lack of efficacy and/or side effects (including cognitive decline) [14]. Whether these side effects are due to on- or off-target effects of the BACE1 inhibitors is not known. However, BACE1 is responsible for cleavage of other substrates as well, and knockout of BACE1 in mice caused both physiological and behavioral deficiencies [14]. These included increased astrogenesis, impaired axonal structure, impaired neuronal maturation and migration, impairments in long-term potentiation (LTP) and long-term depression, as well as cognitive and emotional memory deficiencies [14].

Although certain variants of Aβ peptides are considered toxic, they are endogenously produced by neurons [15] and have been suggested to be involved in neuronal development and differentiation, as well as neuronal function [16–19]. Therefore, a partial reduction of Aβ instead of aiming for complete removal or high-grade inhibition could have better outcome. It is thus of great importance to investigate how a reduction in Aβ by novel AD drugs affects synaptic function, before proceeding to clinical trials. In this study, we applied an established method to measure synaptic activity [20] to investigate if BACE inhibition could reduce Aβ without affecting synaptic transmission. The optical electrophysiology platform has previously been used to investigate the effects on synaptic transmission of, e.g., mood disorder drugs, such as the N-methyl-d-aspartate (NMDA) receptor antagonist dizocilpine and the anticonvulsant NBQX, which is an antagonist at α-amino-3-hydroxy-5-methyl-4-isoxazolepropionic acid (AMPA) receptors [21]. Here, it was used to screen for effects on synaptic transmission following treatment with three BACE inhibitors: BACE inhibitor IV, LY2886721, and lanabecestat. BACE inhibitor IV has been shown to decrease Aβ levels in cell-conditioned media [22], but has not been tested in clinical trials. LY2886721 is a selective BACE inhibitor, which does not affect the other aspartyl proteases and is known to decrease both brain amyloid deposition and cerebrospinal fluid (CSF) Aβ levels [23]. It was the first BACE inhibitor to reach a phase II clinical trial, but the trial was halted due to liver toxicity [24]. Lanabecestat (also called AZD3293 or LY3314814) is a powerful BACE1 inhibitor, known to reduce Aβ in CSF as well as to reduce Aβ positron emission tomography (PET) imaging in the brain of both healthy individuals and AD patients [25]. Lanabecestat was tested in clinical trials, but was withdrawn due to lack of efficacy, and it has also been suggested to worsen memory [26].

All three BACE inhibitors act by binding to the active site of BACE1 and BACE2 and were chosen based on their activity on BACE1/2 or outcomes from the clinical trials. BACE inhibitor IV is more selective to BACE1 than BACE2 [27], but did not reach clinical trials. Both LY2886721 and lanabecestat have been tested clinically, in phase I/II and II/III trials respectively (ClinicalTrials.gov NCT01561430) [26]. They have been shown to inhibit BACE1 and BACE2 equally and the doses used in clinical trials were comparable, but the reported side effects differed. Therefore, we wanted to compare in vitro effects on synaptic transmission between these inhibitors.

We aimed to investigate if BACE inhibitors would affect synaptic transmission at inhibition levels causing clinically relevant Aβ reduction and, if so, if Aβ could be partially decreased through BACE inhibition, as observed in patients with the protective Icelandic mutation [11], without affecting synaptic transmission. We found that all BACE inhibitors investigated affected synaptic transmission negatively at high concentrations, but that reduced transmission as a result of BACE inhibition could not be explained solely by reduced Aβ levels. Low-dose BACE inhibition resulting in moderate Aβ reduction (30–50%) did not affect synaptic transmission for any of the inhibitors tested.

## Materials and methods

### Cell culturing and treatment

Cortical neurons from E18 Sprague Dawley rats were obtained as described previously [28]. The cortical tissue was gently triturated with a sterile, silanized glass Pasteur pipette in Hibernate E to dissociate the tissue. The solution was left for 1 min to precipitate the non-dissociated tissue, and the supernatant from each tube was then transferred and pooled in a 15-ml tube. After trituration, the cell suspension was spun down for 5 min at 250×*g* and the pellet was carefully re-suspended with the silanized Pasteur pipette. NBActiv4 with gentamycin sulfate (10 μg/ml) was added to the cell suspension, and cells were carefully triturated to dissociate cell aggregates. The cell suspension was strained on a 40-μM pore diameter cell strainer to reduce the amount of larger pieces of tissue, and cells were counted using a Scepter cell counter. One hundred thousand cells/well were added per well in poly-d-lysine-coated 96-wells plates. The plates were incubated at 37 °C, 5% CO_2_, and 95% humidity for 14 days to allow for formation of synaptically connected neuronal networks.

On 10 days in vitro (DIV), cells were exposed to either the BACE inhibitors LY2886721 (S2156; Selleckchem), BACE inhibitor IV (565,788; Merck Millipore) or lanabecestat (S8193; Selleckchem), or the γ-secretase inhibitor LY411575 (S2714; Selleckchem), or the α-secretase ADAM10 inhibitor GI254023X (also inhibiting MMP9 and MMP13) (S8660; Selleckchem), or a vehicle control (DMSO) for 4 days. All compounds were added at three concentrations: 0.04, 0.3, and 3 μM final concentration. All procedures for experiments were performed in accordance with the ethical committee in Gothenburg (ethical permit 5.8.18-11305/2018) and followed the guidelines of the Swedish National Board for Laboratory Animals.

### Compound effects on Aβ secretion

Cell medium was collected and pooled from wells previous to stimulation, centrifuged at 400×*g* and stored at − 80 °C until further analysis. Aβ concentrations in conditioned media were measured using the 4G8 Aβ Triplex kit (Mesoscale Diagnostics, Rockville, MD) according to the manufacturer’s instruction. Assay plates were analyzed using QuickPlex SQ 120 instrument and Discovery Workbench software (Meso Scale Discovery).

### Compound effects on synaptic transmission

**Fig. 1:**
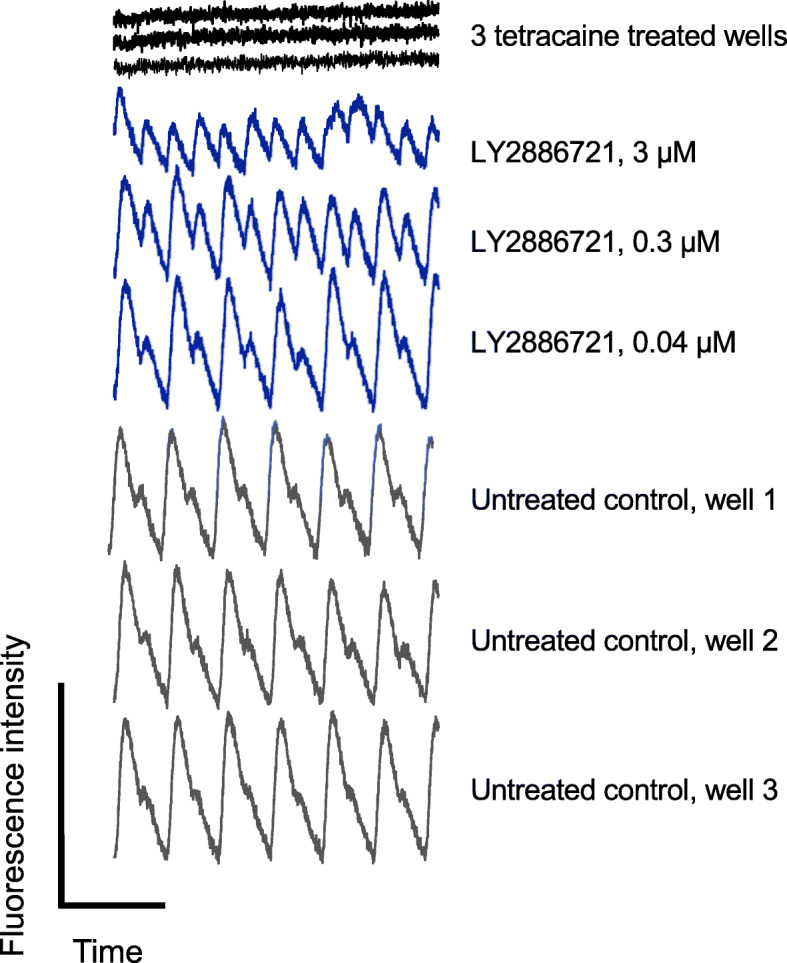
Example traces of calcium fluorescence intensity on the optical electrophysiology platform. The traces represent example concentration-dependent changes in the amplitude of calcium fluorescence intensity as a result of electric field stimulation (EFS) on an optical electrophysiology platform for three concentrations of LY2886721 (blue) and control wells (untreated control: grey; tetracaine-treated wells (30 μM): black). *Y*-axis: fluorescence intensity (bar equals 100 relative fluorescence units). *X*-axis: time (bar equals 1 min)

Four days after compound addition to the cortical cultures (14 days after seeding), electric field stimulation (EFS) experiments were performed on an optical electrophysiology platform (Cellectricon AB, Mölndal, Sweden) [20], where the effect of the compounds on synaptic transmission was investigated. Before the EFS experiments, a calcium indicator (Calcium 5, Molecular Devices, CA, USA) was added to the neuronal cultures for one hour. The cell plates were then inserted into the optical electrophysiology platform and an EFS protocol was subsequently applied. After 14 days, neurons in the cortical cultures were synaptically connected and the response to EFS was reproducibly transduced to areas distant from the stimulating electrodes. To enable quantification of changes in the synaptic signal caused by EFS, the fluorescence intensity from the calcium probe was simultaneously monitored in regions of interest (ROIs) distant to the stimulation electrodes. The measured signal was derived from neurons that were synaptically connected to the neurons stimulated by the electric field. Reference compound tetracaine was also included as a positive control on each plate and for normalization purposes (the effect of a high concentration of tetracaine fully blocks the synaptic calcium response and this response was set to 0%). DMSO control wells were also present on each plate (same [DMSO] as in the drug-treated wells), and the resulting calcium response in the control wells was set to 100% during the normalization. To ensure consistency between preparations, the reference compound allopregnanolone (a positive allosteric GABAA modulator) was also present in concentration-response format. The effect of the compounds on the synaptically mediated increases in Ca^2+^ fluorescence on the optical electrophysiology platform [20] was then quantified: The normalized median value of the approximately 12 pulses that were captured during a 3-min EFS train (0.33 Hz) is presented. See Fig. 1 for example calcium fluorescence intensity traces for untreated control wells, tetracaine-treated wells, and LY2886721 from one of the two individual primary cortical cultures.

### Statistical analysis

Mean values from separate experiments (*n*) were compared using Student’s two-tailed *t* test. Statistical significance was defined as *p* < 0.05. All statistical analyses were performed using GraphPad (Prism version 7.02 for Windows, GraphPad Software, La Jolla, CA, USA, www.graphpad.com).

## Results

### The effect of BACE inhibitors on Aβ secretion and synaptic transmission

**Fig. 2:**
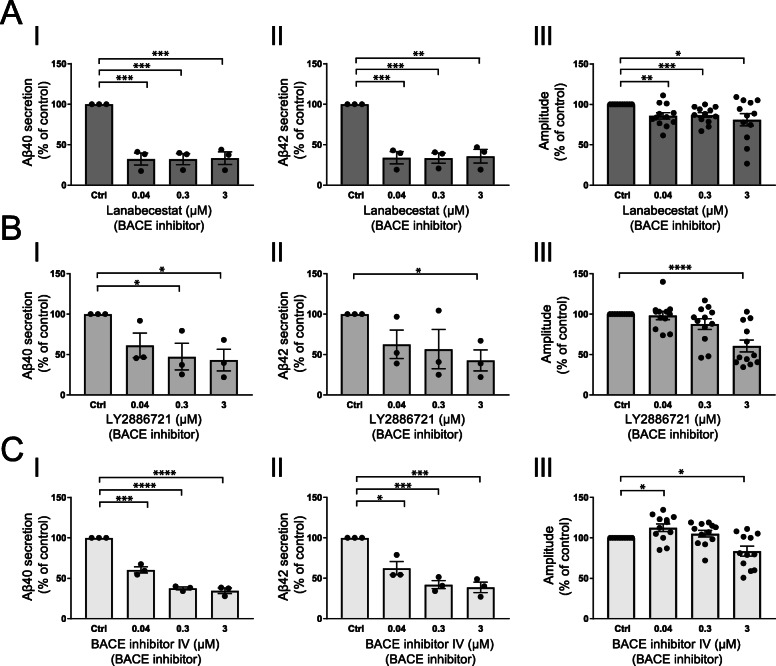
Amyloid β secretion and synaptic transmission upon β-secretase inhibition. Primary rat neurons treated with 0, 0.04, 0.3, and 3 μM of three different BACE inhibitors for 4 days. Thereafter, Aβ secretion to the cell-conditioned media was measured using immunochemiluminescence techniques. Synaptic transmission was measured using an optical electrophysiology platform. **a** All concentrations tested for lanabecestat equally decreases Aβ40 (I) and Aβ42 (II). All concentrations also decrease synaptic transmission between neurons (III). **b** While 0.04 μM LY286721 does not affect Aβ40 secretion, both 0.3 μM and 3.0 μM decreases secretion of Aβ40 (I). Of the three doses tested, only 3.0 μM LY286721 significantly decreases Aβ42 (II) and decreases synaptic transmission between neurons (III). **c** Already 0.04 μM BACE inhibitor IV decreases secretion of Aβ40 (I) and Aβ42 (II) and both 0.3 μM and 3.0 μM BACE inhibitor IV decreases the secretion further (III). Whereas 0.04 μM BACE inhibitor IV increases synaptic transmission, 0.3 μM has no effect and 3.0 μM decreases synaptic transmission. Both secretion and synaptic transmission normalized with DMSO control and presented as percentage. Bars represent mean +/− SEM. *n* = 3 from 3 individual primary cortical cultures for secretion data, *n* = 12 separate experiments from 2 individual primary cortical cultures for synaptic transmission data. Student’s *t* test is performed between the controls and each concentration. *p ≤ 0.05, **p ≤ 0.01, *** *p* ≤ 0.001, ****p ≤ 0.0001

To investigate the effect of the three different BACE inhibitors on synaptic transmission, we treated cortical rat neuronal cultures with the BACE inhibitors for 4 days and thereafter investigated Aβ secretion and synaptic transmission. Based on previous publications, where low μM concentrations of the BACE inhibitors were used in vitro [22, 29], the concentrations were titrated down from 3 to 0.04 μM, aiming to induce partial BACE inhibition. Three micromolar resulted in high Aβ reduction, similar to those observed in clinical trials [30]. Lanabecestat treatment significantly decreased secretion of both Aβ40 (Fig. 2a (I)) and Aβ42 (Fig. 2a (II)) with approximately 70% at all concentrations tested. Correspondingly, all three concentrations decreased synaptic transmission with approximately 14–18% (Fig. 2a (III)).

Treatment with 0.04 μM LY2886721 resulted in a trend towards decreased secretion of Aβ40 with 39%, while 0.3 μM significantly decreased Aβ40 with 53% and 3 μM decreased Aβ40 with 57% (Fig. 2b (I)). LY2886721 treatment also showed a trend towards decreased secretion of Aβ42 with 37% at 0.04 μM and with 43% at 0.3 μM and significantly decreased Aβ42 with 57% at 3 μM (Fig. 2b (II)). LY2886721 at 0.04 μM and 0.3 μM did not affect synaptic transmission, while 3-μM LY2886721 treatment significantly decreased synaptic transmission with 39% (Fig. 2b (III)).

BACE inhibitor IV treatment of primary neurons in culture significantly decreased secretion of Aβ40 with 39% at 0.04 μM, with 62% at 0.3 μM and with 65% at 3 μM (Fig. 2c (I)). Similarly, BACE inhibitor IV significantly decreased secretion of Aβ42 with 38% at 0.04 μM, with 58% at 0.3 μM and with 61% at 3 μM (Fig. 2c (II)). Investigation of synaptic transmission revealed that treatment with 0.04 μM BACE inhibitor IV resulted in a small but significant increase in synaptic transmission (13%), while 0.3 μM showed no affect and 3 μM significantly decreased synaptic transmission with 16% (Fig. 2c (III)).

### The effect of γ-secretase and α-secretase inhibitors on synaptic transmission and Aβ secretion

**Fig. 3:**
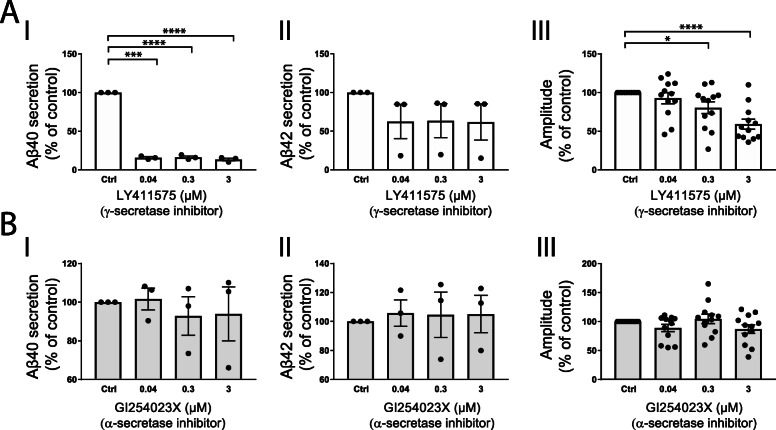
Amyloid β secretion and synaptic transmission upon γ- and α-secretase inhibition. Primary rat neurons were treated with 0, 0.04, 0.3, and 3 μM γ-secretase inhibitor LY411575 or ADAM10 inhibitor GI254023X for 4 days. Secreted Aβ40 and Aβ42 to the cell-conditioned media were measured using immunochemilumisence techniques. Synaptic transmission was measured using an optical electrophysiology platform. **a** All three concentrations decrease secretion of Aβ40 (I) into cell culture media with approximately 80%. All three concentrations tended to decrease secretion of Aβ42 with approximately 40% (II), although not reaching statistical significance. Synaptic transmission (III) was significantly decreased when cells were exposed to 0.3 μM LY411575, and the transmission was further decreased at 3 μM. **b** GI254023X did not affect secretion of Aβ40 (I) or Aβ42 (II), nor did it have any effect on synaptic transmission (III). Bars represent mean +/− SEM. *n* = 3 from 3 individual primary cortical cultures for secretion data, *n* = 12 separate experiments from 2 individual primary cortical cultures for synaptic transmission data. Student’s *t* test is performed between the controls and each concentration. *p ≤ 0.05, ***p ≤ 0.001, ****p ≤ 0.0001

To further test if synaptic transmission was related to inhibitor effects on Aβ secretion, we investigated inhibitors of other secretases involved in APP processing. The neurons were treated with one γ-secretase inhibitor (LY411575), expected to decrease both Aβ40 and Aβ42, and one α-secretase inhibitor (the selective ADAM10 inhibitor GI254023X) that cleaves APP in the middle of the Aβ sequence and is not expected to affect the production of Aβ40 and Aβ42 [4] as described above, before measuring synaptic transmission. As predicted, γ-secretase inhibition with LY411575 decreased Aβ40 secretion with about 85% at all concentrations tested (Fig. 3a (I)) and, although not statistically significant, a trend towards decrease (about 37%) in Aβ42 secretion was observed at all concentrations (Fig. 3a (II)). While 0.04-μM LY411575 treatment did not affect synaptic transmission, it significantly decreased synaptic transmission with 20% at 0.3 μM (Fig. 3a (III)) and with 41% at 3 μM. The ADAM10 inhibitor GI254023X did not, as expected, affect secretion of Aβ40 (Fig. 3b (I)) or Aβ42 (Fig. 3b (II)), and no effect on synaptic transmission was observed (Fig. 3b (III)).

## Discussion

So far, all clinical trials involving β-secretase inhibitors have been terminated due to side effects and/or lack of positive effects, and some studies even report cognitive decline possibly due to negative effects on putative physiological functions of APP and/or Aβ. Here, we evaluated the effects of three BACE inhibitors on Aβ secretion and synaptic transmission, using electrochemiluminescent measurement techniques and an optical electrophysiology platform.

We found that negative effects of BACE inhibition on synaptic transmission did not correspond dose-dependently with decreased Aβ secretion. Instead, all three inhibitors tested affected synaptic transmission negatively, but only at concentrations that decreased Aβ42 secretion by at least 50%. For example, all concentrations of lanabecestat decreased the secretion of both Aβ40 and Aβ42 by more than 50% and, consistently, all concentrations of lanabecestat also decreased synaptic transmission. This is in concordance with previous publications showing that long-term exposure of high-dose BACE inhibitors reduced spine density in hippocampal neurons of mice [31, 32] and weakened synaptic transmission [31, 33].

The only concentration of LY2886721 that decreased synaptic transmission was the highest concentration of 3 μM. While all concentrations decreased Aβ40 with 50% or more, 3 μM was the only concentration of LY2886721 that decreased secretion of Aβ42 with 50%. This highlights the importance of future studies addressing possible links between Aβ42 and synaptic transmission. Although it is difficult to compare in vivo doses to the in vitro equivalents, it is somewhat surprising that all three doses of lanabecestat resulted in the same, high levels of Aβ inhibition. LY2886721 and lanabecestat were used at similar doses in the clinical trials (between 15 and 70 mg daily for LY2886721 and 20 and 50 mg daily for lanabecestat) with comparable effects on Aβ reduction (between 50 and 70% in CSF, depending on dose) [26].

BACE inhibitor IV decreased Aβ secretion at all three concentrations, but only the highest concentration, decreasing Aβ secretion by 60%, affected synaptic transmission negatively. In fact, the lowest concentration with a mild effect on Aβ secretion (40% decrease) even increased synaptic transmission compared with control. These results are consistent with previous studies showing that BACE^−/−^ mice have alterations in LTP [34] and hippocampus-dependent cognition [35] whereas BACE^+/−^ mice (only reducing BACE activity by 50%) do not. Still, the fact that treatment with 0.3 μM BACE inhibitor IV did not affect synaptic transmission, even though Aβ levels were decreased by more than 50%, shows that the effects of BACE inhibition on neuron communication is not only dependent on Aβ levels, but also other factors may contribute. A relatively recent clinical trial conducted with a BACE inhibitor showed that inhibiting BACE increased sAPPα in CSF (ClinicalTrials.gov: NCT01827982 and NCT01887535) [36, 37], possibly as a results of more APP available as substrate for α-secretase cleavage. sAPPα has been shown to be neuroprotective and to have positive impact on cognitive functions [38]. Although the reason behind the increased synaptic transmission by the lowest dose of BACE inhibitor IV in our study must be further investigated, moderate inhibition of BACE could potentially increase α-cleavage and thus indirectly have a positive effect on synaptic activity. Additionally, BACE inhibitor IV is the only inhibitor tested here that is more selective to BACE1 than to BACE2 [27]. Hence, the increase in synaptic transmission with the lowest dose (0.04 μM) could possibly also be a result of BACE inhibitor IV affecting BACE1 specifically while sparing BACE2 activity, although the BACE2 effect is unknown.

Although the Aβ reduction could be responsible for at least some of the negative effects on synaptic transmission, BACE1 has several other substrates in addition to APP (reviewed in [39]), some of which are involved in dendritic spine dynamics, synaptic transmission, and memory formation [40], including the seizure-related gene 6 (SEZ6), close homolog of L1 (CHL1), and neuroligin 1 (NLGN1) proteins. Inhibiting BACE1 has been shown to reduce spine density in hippocampal neurons through SEZ6 [31, 32] and BACE null mice display axonal growth and myelination deficiencies, leading to abnormal or decreased synaptic transmission, due to altered cleavage of CHL1 and NLGN1 [41–43]. However, the alterations that are linked to SEZ6, CHL1, and NLGN1 proteins have been observed upon long-term inhibition of BACE1 or in BACE1 knockout mice [31, 32, 41–43]. In the current study, cells were treated with BACE inhibitors at a single occasion and the observations were made 4 days post-treatment in order to investigate if Aβ can be reduced without affecting synaptic transmission. The involvement of other BACE substrates in altered synaptic transmission following short, low-dose BACE inhibition would be interesting to examine in future studies. In line with this, it would also be interesting to explore if combinations of low-dose BACE inhibitors could result in additive effects on Aβ reduction without causing additive synaptic dysfunction.

Inhibition of γ-secretase using LY411575 decreased secretion of both Aβ40 and Aβ42 equally at all three doses, although the decrease of Aβ42 did not reach statistical significance. The preferential inhibition of Aβ40 over Aβ42 has earlier been shown for other γ-secretase inhibitors, by our group and others [44, 45]. This biased inhibition results in an increased Aβ42 to Aβ40 ratio, which is believed to be more amyloidogenic and could be negative for synaptic function [46]. Indeed, we saw a dose-dependent decrease in synaptic transmission upon treatment with the γ-secretase inhibitor LY411575. Inhibiting α-secretase using GI254023X did not affect secretion of Aβ and had no effect on synaptic transmission.

## Conclusion

In conclusion, we show that the optical electrophysiology platform is a suitable system to test possible side effects of drugs targeting neurodegeneration before reaching clinical trials and demonstrate that BACE inhibitor concentrations resulting in decreased Aβ levels corresponding to the ~ 30% reduction seen in carriers of the protective Icelandic mutation [12] do not affect synaptic transmission negatively. We therefore suggest that clinical studies should aim for a moderate central nervous system exposure of BACE inhibitors to avoid side effects caused by effects on synaptic efficacy.

## Data Availability

The datasets used and/or analyzed during the current study are available from the corresponding author on reasonable request.

## Abbreviations

Aβ: Amyloid beta
AD: Alzheimer’s disease
AMPA: α-Amino-3-hydroxy-5-methyl-4-isoxazolepropionic acid
APP: Amyloid beta precursor protein
BACE: Beta secretase
CHL1: Close homolog of L1
CSF: Cerebrospinal fluid
DIV: Days in vitro
EFS: Electric field stimulation
FAD: Familial Alzheimer’s disease
LTP: Long-term potentiation
NLGN1: Neuroligin 1
NMDA: N-Methyl-d-aspartate
PET: Positron emission tomography
SEZ6: Seizure-related gene 6
